# Measuring EGFR Separations on Cells with ∼10 nm Resolution via Fluorophore Localization Imaging with Photobleaching

**DOI:** 10.1371/journal.pone.0062331

**Published:** 2013-05-01

**Authors:** Sarah R. Needham, Michael Hirsch, Daniel J. Rolfe, David T. Clarke, Laura C. Zanetti-Domingues, Richard Wareham, Marisa L. Martin-Fernandez

**Affiliations:** 1 Central Laser Facility, Research Complex at Harwell, STFC Rutherford Appleton Laboratory, Harwell Oxford, Didcot, Oxfordshire, United Kingdom; 2 Department of Engineering, University of Cambridge, Cambridge, United Kingdom; University of Iowa, United States of America

## Abstract

Detecting receptor dimerisation and other forms of clustering on the cell surface depends on methods capable of determining protein-protein separations with high resolution in the ∼10–50 nm range. However, this distance range poses a significant challenge because it is too large for fluorescence resonance energy transfer and contains distances too small for all other techniques capable of high-resolution in cells. Here we have adapted the technique of fluorophore localisation imaging with photobleaching to measure inter-receptor separations in the cellular environment. Using the epidermal growth factor receptor, a key cancer target molecule, we demonstrate ∼10 nm resolution while continuously covering the range of ∼10–80 nm. By labelling the receptor on cells expressing low receptor numbers with a fluorescent antagonist we have found inter-receptor separations all the way up from 8 nm to 59 nm. Our data are consistent with epidermal growth factor receptors being able to form homo-polymers of at least 10 receptors in the absence of activating ligands.

## Introduction

Epidermal growth factor receptor (EGFR) is the founding member of a family of four homologous receptor tyrosine kinases (EGFR/HER2-4, ErbB1-4), which are initiators of signals for cell proliferation, survival, and transformation [1]. The extracellular domain of the EGFR is comprised of four subdomains (I to IV). The unliganded receptor monomer is held in a closed conformation by an intramolecular tether formed by loops in subdomains II and IV [2]. In ligand-occupied receptor dimers, the intramolecular tether is broken, and the receptor is opened into an extended conformation which interacts with another monomer, forming a back-to-back dimer [3], [4]. Ligand-induced receptor dimerisation is thought to be the key stimulatory step, leading to the formation of an asymmetric dimer [5] and allosteric transactivation of the two associated intracellular EGFR kinases. There is mounting evidence, however, that unliganded receptors may already be dimerised or oligomerised before binding agonist ligand [6], [7], [8], [9], [10], [11], and that EGFR activation may also be facilitated/regulated by receptor confinement within plasma membrane domains (e.g. lipid-rafts and/or membrane skeleton fences) [12], [13], [14], [15]. However, it has also been suggested that the dimerisation and clustering of inactive EGFRs may be an artifact of overexpression, and that inactive receptors are monomeric when expressed at low physiological levels [16]. Defining the oligomerisation state of inactive receptors is crucial to unravel the roles of ligand binding, receptor dimerisation, confinement and clustering, all of which are still poorly understood. However, the lack of methods with sufficient resolution has hindered these investigations.

Detecting receptor dimers and distinguishing them from other forms of clustering requires access to techniques that can provide distance information in a range that includes molecular length scales (1–20 nm) and the putative scale of plasma membrane nanodomains (20–100 nm) [17]. Measuring these distances, however, poses significant challenges. Electron paramagnetic resonance spectroscopy [18] and fluorescence resonance energy transfer (FRET) [19] can measure the relative separations of magnetic and fluorescence probes bound to sites of interest in the same protein or in different proteins, and are widely used to study protein interactions in the range ∼2–8 nm; however, this range is smaller than many transmembrane protein dimers, including EGFR dimers. Clusters of assemblies are intractable for X-ray crystallography [20]. Immuno-electron microscopy [14] and atomic force microscopy [21] typically require the use of antibodies for membrane protein recognition and this, among other reasons, limits their resolution to a few tens of nanometres. Optical super-resolution imaging methods such as photoactivated localisation microscopy (PALM) [22], stochastic optical reconstruction microscopy (STORM) [23], [24], stimulated emission depletion (STED) [25] and near-field scanning optical microscopy (NSOM) [26] are typically limited to resolutions of >20–30 nm and cannot be used to measure inter-molecular distances. As a result, inter-molecular distances in the range of ∼10–50 nm remain completely uncharted.

Absolute molecular positions can be derived using single-fluorophore localisation, in which the position of a fluorescent molecule is determined by fitting a model profile to its diffraction-limited image spot [27]. Localisation precision is limited by the signal-to-noise ratio (SNR), but 1–2 nm has been achieved. The combination of fluorophore localisation imaging with photobleaching (FLIP) is the foundation of techniques that can achieve a resolution of <10 nm in the *x, y* plane, such as single-molecule high-resolution imaging with photobleaching (SHRImP) [28] and nanometer-localized multiple single-molecule (NALMS) microscopy [29]. These methods measured separations of around ∼10 nm between fluorophores bound to a glass-immobilised double-strand DNA molecule from the shift in the centroid position of a diffraction-limited spot that follows independent quantal photobleaching of the fluorophores. However, the application of methods like SHRImP and NALMS to the measurement of population-averaged separations has so far been restricted to the measurement of single distances in monodisperse DNA oligos. Key challenges of applying these methods to cells include the presence of inhomogeneous populations separated by an unknown number of distances that have to be resolved. Other challenges are the additional noise contributions from autofluorescence background, which is reduced but not eliminated by the use of total internal reflection fluorescence (TIRF) microscopy [30], and the intrinsically wider spread of SNRs, as TIRF exposes probes in the plasma membrane at varied axial distances to different excitation field strengths. Here we extend the FLIP concept to the cell environment and demonstrate its usefulness by measuring the separations between fluorescent antagonist-labelled EGFR molecules on the surface of unstimulated T47D cells, a breast cancer cell line that express the receptor at low physiological levels (∼7,000 copies per cell [31], [32]). Given that the notional separation resolving power of the method depends on the accuracy with which the errors are estimated, critical for success was the development of a set of diagnostics based on error analysis that ensured that the data were not over-interpreted.

## Results

### Determination of the Separation between Two Molecules

**Figure 1:**
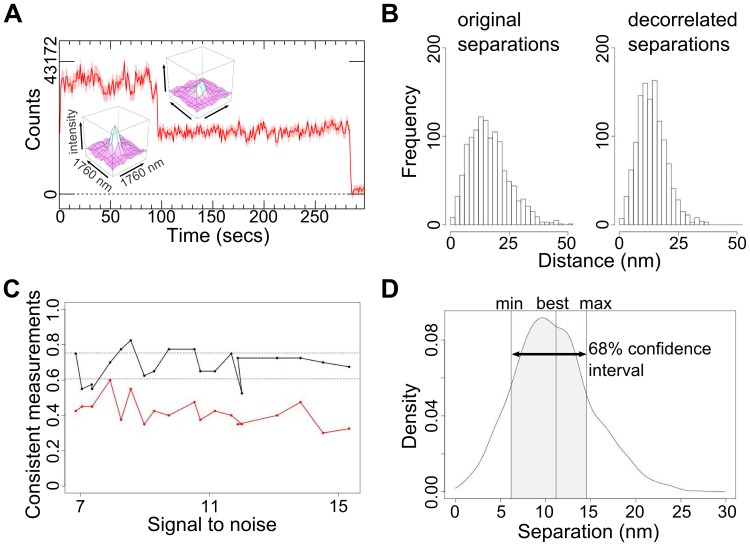
Measuring the separation between two molecules. (A) Example intensity v time course from a spot on cells showing a pair of Affibody-Atto 647N molecules photobleaching in two steps. Images were acquired every 0.28 s. The insets show the profile of the spot before and after the first bleaching event. (B) Original and decorrelated separations to illustrate the effect of anti-correlated positional error. The data of each spot is resampled and fitted 1200 times yielding 1200 sets of seven parameters that are samples of the 7-dimensional parameter distribution. In the decorrelated version the parameters have been independently randomly reordered, i.e. parameters from the same set end up in different sets. (C) Comparison of the error estimation of the Thompson, Larson and Webb [34] and sequential photobleaching NALMS analysis method [29] (red line) to our FLIP variant (black line) for synthetic data, depending on the SNR. SNR is the ratio of the measured mean intensity of a single-fluorophore trace to its standard deviation. The graph shows how often the real distance lies within the 68% confidence interval of the measurement. For [34], the confidence interval was taken as separation estimate ± standard deviation; for FLIP the confidence interval was taken as the shortest interval that contains 68% of the separation results from the bootstrap sampling (1,200 samples per measurement). (D) A probability density plot of the separations from the bootstrap samples of an individual measurement, showing the best fit value and the confidence interval.

Like in SHRImP and NALMS, the distance between fluorophores was derived from the change in the diffraction-limited image spot when one of them photobleaches. First, spots in single molecule images were identified and their intensities tracked through time using custom Bayesian algorithms, which are best suited to the poor SNR typical of the cell environment [33]. We selected spots with two constant intensity levels separated by abrupt photobleaching steps where the lowest level decays to zero (negative intensities indicate background interference) and with more than 5 frames per level (Fig. 1A). The latter set a threshold in the number of photons and a therefore minimum localisation precision. If blinking occurred, areas between blinking steps were also used if the intensity between them remained constant. To each selected spot the FLIP algorithm fits a model of two stationary fluorophores, each with constant intensity. We assumed two 2D Gaussian profiles of identical fixed full width at half maximum (FWHM) (the point spread function (PSF) of a single emitter is an Airy disk, but the differences with a Gaussian function are minor [28], [33]). We allowed for uniform backgrounds in the fitted region around the spot that are different in every frame. Like SHRImP, we used a global least-squares 7-parameter fit to identify the best intensity, *x* and *y* positions and PSF FWHM for each fluorophore (one PSF at FWHM is about 430nm). We found this approach to be less sensitive to anticorrelated positional errors than the sequential fitting of levels and subtraction procedure of NALMS, which can relocate the two fluorophores in opposite directions while maintaining the same centroid position. From simulations, this effect is more pronounced for short distances, when the uncertainty in the separation is significant compared to its value and can make distances appear smaller than the original value (Fig. 1B). Our simulations also showed that the commonly used localisation precision estimate [34] systematically underestimates errors (Fig. 1C). We therefore assessed the probability distribution of the model parameters using the Monte Carlo bootstrap method [35]. This inherently accounts for all noise components including background. The *x* and *y* positions fitted to the bootstrap-resampled datasets were converted to a distribution of separations (Fig. 1D). We defined the best estimate of the separation (i.e. the best fit) as the least-squares fit value to the true (unresampled) datasets and the associated 68% confidence interval (CI) as the interval that contains 68% of the bootstrap separation samples nearest to the best fit value (see Materials and Methods). As separations cannot be negative, the distribution is an asymmetric Rice-like distribution [36], instead of a Gaussian, but 68% would be within ±1standard deviation (*σ*) if it were. Using simulations, we found that this approach provided accurate errors (Fig. 1C). As bootstrapping can be computationally demanding, we used Graphics Processing Units to maximize speed.

### Correcting for Sample Drift

**Figure 2:**
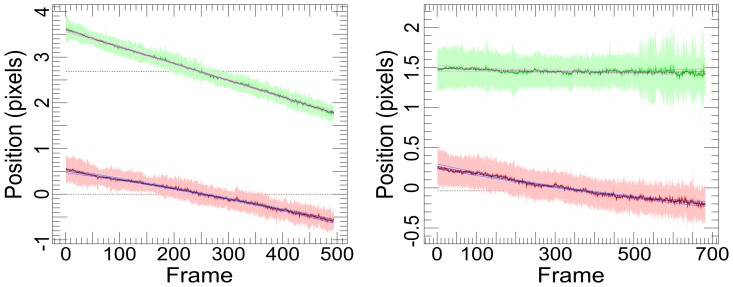
Examples of observed large (left) and small (right) sample drifts. The plots show a combined aligned track for the drift in position (pixels) in X (solid red line) and Y (solid green line) from all tracks in a set of images. The fitted global drift of the sample, a quadratic model fitted using ordinary least squares, is shown as a blue line for X and lilac for Y.

We observed sample drifts in the range of ∼0.03–2 nm/s. Drift problems have been previously reported [22] and most are caused by temperature variations. To compensate, we superimposed all the tracks in each set of images and performed a least-squares fit of a quadratic model to their mean track, which represents the global drift of the sample (Fig. 2), and was corrected for during the fitting process. We speculate that SHRImP and NALMS were not affected by drift because of the much faster data accumulation rates afforded by high power lasers [28] and the increased stability of the custom microscopes used [29]. By using a commercial TIRF microscope with a temperature-controlled enclosure and considering drift in our analysis, we have ensured that our method is accessible to most cell biologists.

### Building Population-averaged Separation Distributions in Cells

**Figure 3:**
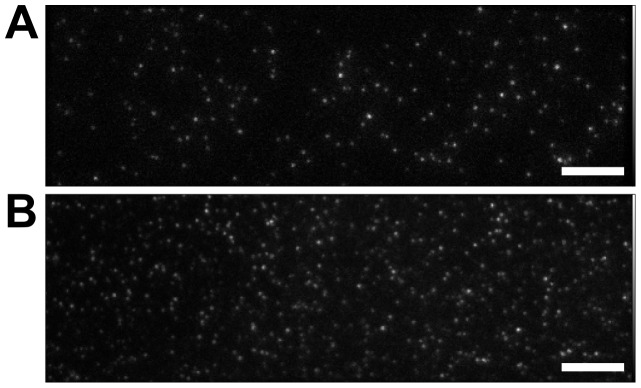
Comparing single molecule TIRF images from glass-immobilised molecules and from cells. (A) Single-molecule TIRF image of glass-immobilised 13.3 nm DNA rulers, labelled at each end with Atto 647 N. (B) Single-molecule TIRF image of T47D cells labelled with anti-EGFR Affibody-Atto 647 N. Scale bar = 8 µm.

**Figure 4:**
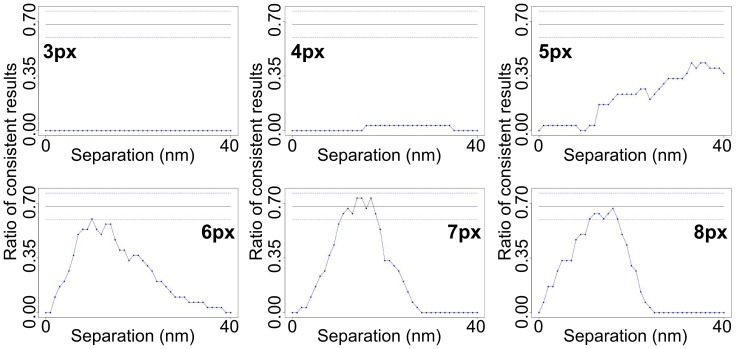
How close a second spot can be without affecting the calculated separation. Simulations to determine how close, using distances in the range 3–8 pixels (as indicated to the left or right of each plot), a second spot can be before it affects the calculated separation within the first spot. If it is closer than 7 pixels (FWHM of the spot is 3 pixels), the separation is miscalculated and the percentage of consistent measurements does not reach 68%. Each plot shows data from 30 tracks. The simulated separation is 13 nm.

**Figure 5:**
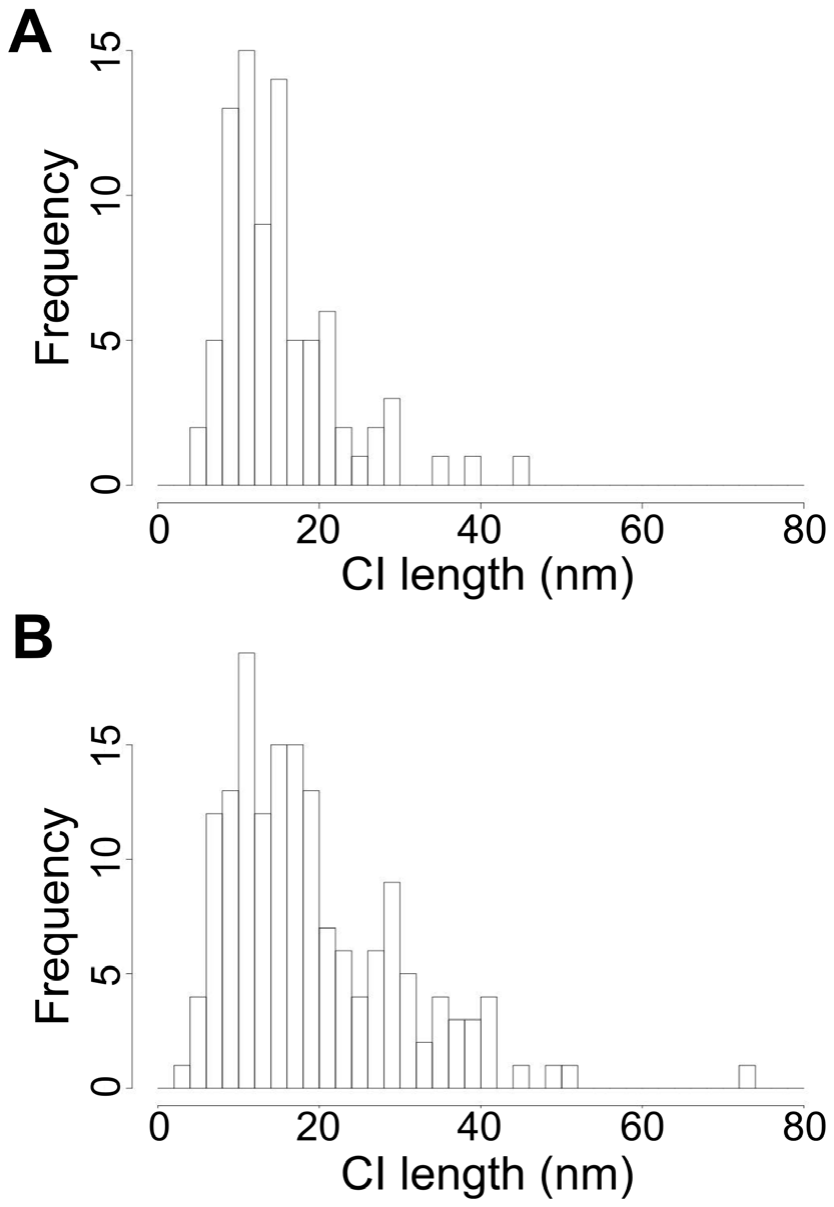
Distribution of the length of the confidence intervals. (A) From experimental data from the 13.3 nm DNA ruler immobilised in glass. (B) From EGFR-Affibody on the surface of T47D cells.

**Figure 6:**
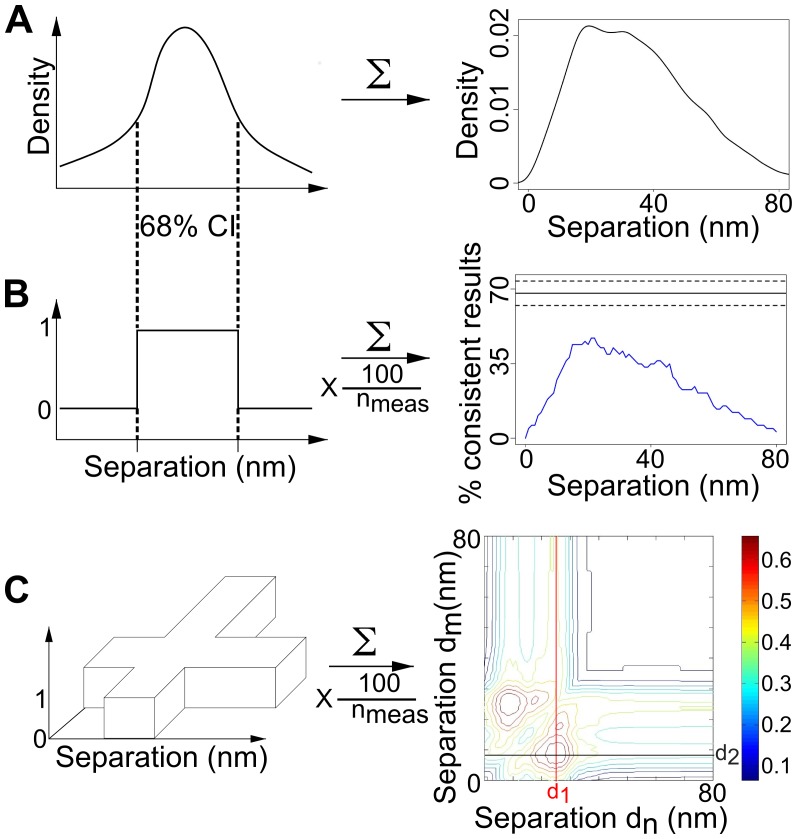
Using confidence intervals as diagnostics. (A) A sum of the individual bootstrap separation densities. The left-hand side shows a cartoon of the separation distribution of a single two-fluorophore spot. The right-hand side shows the distribution of the combined separation data from multiple two-fluorophore spots. (B) A _1s_CI-Plot (right) is the scaled sum of top hat functions (left). Each top hat is created from the bootstrap separation distribution of a two-fluorophore spot. The top hat - or indicator - function has the value 1 if the separation lies within the 68% confidence interval, otherwise it has the value 0. The _1s_CI-Plot plot (right) is an example sum of such functions for multiple two-fluorophore spots, where the scaling consists of dividing by the number of spots and then multiplying by 100 for a % scale. Hence, a value of 50% at a particular separation means that separation lies in the 68% confidence interval of the separation distribution of half of the two-fluorophore spots included in the analysis. The black line shows the ideal peak value (68%) expected for a repeated measurement of a single distance and the dashed lines show the uncertainty of this ideal due to the limited number of samples. (C) A _2s_CI-Plot is the scaled sum of crosses, all with the same height and with equal arm widths given by the 68% confidence limits of each individual bootstrapped separation.

There are two main challenges to extend FLIP methods (like SHRImP and NALMS) from glass-immobilised biomolecules (Fig. 3A) to cells (Fig. 3B). The first challenge is the intrinsic characteristics of cell data. One of these is molecular crowdedness. While molecular density can be easily adjusted for glass-immobilised biomolecules so that most spots are separated by >∼3 PSFs (or ∼8 pixels), the limit below which contributions from adjacent spots introduce systematic errors (Fig. 4), in images from cells many spots are closer than 3 PSFs and have to be discarded. A second characteristic is the intensity variation between spots in the plasma membrane at different distances from the glass surface where TIRF occurs. Because of this the variation in SNR ratio in cell images is larger than in those from glass-immobilised biomolecules. A third is the presence of residual autofluorescence background not eliminated by TIRF, while images of glass-immobilised biomolecules have minimal background. As a consequence the SNR of cell images is worse, and therefore the distribution of errors in data from glass-immobilised molecules is narrower (Fig. 5A) than in data from cells (Fig. 5B). SHRImP and NALMS exploited the relative abundance of well-separated spots with good SNR in glass-immobilised biomolecule samples, to achieve high resolution by setting a tight separation error threshold (*σ* <±5 nm) and use histograms of best-fit separations to display population-averaged measurements [28], [29]. Because of the poorer SNR in cells the proportion of data that can be kept after setting an error limit of <±5 nm is significantly smaller than from glass-immobilised molecules. When larger errors can be expected, as in cells, it is advantageous to replace histograms of best fit values by a function that includes full separation density function, e.g. a sum of these functions (Fig. 6A). This is because individual separation measurements are normally asymmetric distributions (we expect Rice-like distributions, see Fig. 1D), and given that the least squares fit is used, best fit values are not necessarily in the centre of the underlying distribution. This sum of densities is effectively a histogram in which the each measurement is spread out according to its uncertainty thus better reflecting the complex dependency on SNR inherent to FLIP-derived population-averaged distributions in cells.

The second challenge is the different nature of the separations to be measured. SHRImP and NALMS demonstrated 10 nm resolution in population-averaged single-distance values from monodisperse DNA oligo populations. In cells we expect distributions that indicate different separations where the associated distance peaks may be unresolved, like for example EGFR separations within dimers (∼11 nm) [3], [4] and confinement regions (<20 nm and above). These separations could in principle be separated by deconvolution - e.g. fitting summed Gaussian functions to the data and solving for 3 *n* parameters (position, width and height) for each peak, where *n* is the number of separations and must be small [37]. However, unless the number *n* of separations is known, the complex dependence on the SNR of FLIP-derived distributions makes their resolution by deconvolution challenging - as it depends on numerous assumptions and requires numerous parameters to be determined and adjusted empirically for each data set. Information on the number *n* of separations can be derived from the CIs of the individual separation density functions (Fig. 1D). By definition, we are 68% confident that the real separation of two fluorophores is within the CI, which can be represented by a top hat function of height = 1 and a width defined by the 68% confidence limits. Summing these functions and multiplying by f = 100/(number of measurements) produces a population-averaged diagnostic, hereafter referred to as single-separation CI-based Plot (_1s_CI-Plot) (Fig. 6B, *right*) with interesting properties, where the value at a particular distance is the percentage of measured separations consistent with that distance.

The height of a _1s_CI-Plot from repeated measurements of a single separation *d_true_* is a measure of CI accuracies. The plot will peak at or close to *d_true_* with the height at *d_true_* following a binomial distribution (we expect *d_true_* to lie within 68% of the CIs and not to lie in 32% of them), i.e. the peak at the true distance has height = 68%± *E*, where *E* is the standard deviation of the distribution. Therefore, if the peak height is >68%, then the CIs are too long and the errors have been overestimated. However, if the peak height is <68%, either the CIs are too short and the errors are underestimated, or the number of separations represented by the data set is larger than 1.

Information on the value of *n* can be derived using CI-Plots of n dimension. For example, a two-separation CI-Plot (_2s_CI-Plot) is formed by summing and scaling by *f* 2D *cruciform* top hat functions (one for each measurement) (Fig. 6C). If there are two true separations, then 68% of measurements will agree with them, both individually and together. Consistent with this the _2s_CI-Plot will show two mirrored 2D peaks that reach 68% at or close to the true separations *d_1_* and *d_2_* (Fig. 6C, *red and black cross-sections*). Finding the number of components in this way can help in the analysis of complex separation distributions where data are scarce and in determining the resolution of the measurement, as we illustrate below using EGFR separations in cells.

### Verifying the FLIP Algorithm using Simulated Data and DNA Rulers

**Figure 7:**
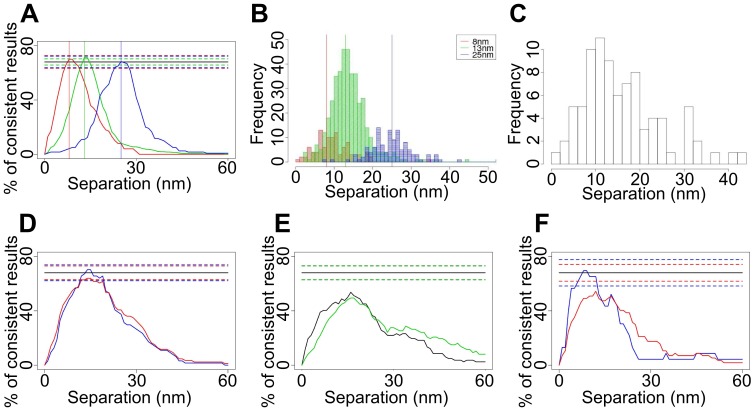
Population-averaged single-distance separations. (A) _1s_CI-Plots for simulated separations of 8 nm (red), 13 nm (green) and 25 nm (blue). The true distances are indicated by vertical lines. (B) Histogram of best fits for the 8, 13 and 25 nm simulated data. The calculated separation values from the best fits are 9.6±0.5 nm, 13.9±0.3 nm and 25.1 nm (C) Histogram of best fits and (D) _1s_CI-Plots for the 13.3 nm DNA ruler data showing data sets where the root mean square of the residuals from fitting to the drift model exceeded 0.04 pixels (6.4 nm) were included (red) and excluded (blue). (E) _1s_CI-Plots for end-to-end distance measurements of 8 nm (black) and 13.3 nm (green) DNA rulers without drift correction. The drift of the sample as a whole makes the distance appear larger and less defined. (F) 8 nm DNA rulers, showing data sets where the root mean square of the residuals from fitting to the drift model exceeded 0.04 pixels (6.4 nm) were included (red) and excluded (blue).

To test the accuracy of the method, we simulated three data sets representing pairs of fluorophores separated by single distances. We chose 8 nm (the upper limit of FRET), 13 nm (comparable to the separations between two 2 nm probes bound to a back-to-back EGFR dimer [3], [4]), and 25 nm (representative of the putative lower bound for the size of confinement regions [17]). We added photon noise and background, and added and corrected for drift. Fig. 7A shows that the peaks of the _1s_CI-Plots are at the correct distances and that the heights are within the 68%±*E* range. We also verified that the algorithm correctly calculated best fit values (Fig. 7B).

To experimentally verify the method, we immobilised 24- and 40-base double helix DNA molecules labelled at both ends with Atto 647N and biotin on streptavidin coated glass surfaces. These oligos are expected to be rigid rods with end-to-end separations of 8 and 13.3 nm [29]. A series of images were taken until most of the fluorophores bleached. As previously observed, many spots showed single bleaching events, presumably because one fluorophore bleached before data collection. Spots were selected using the criteria described previously. In addition, those that moved position at the bleaching step by >^1^/_2_ pixel (80 nm) were also excluded, the latter setting a maximum measurable separation of ∼160 nm. The remaining spots were considered suitable for further analysis if their traces showed at least two intensity levels and at least five frames in each level.

The FLIP algorithm generated a histogram of best fit values for the drift-corrected 13.3 nm ruler data that peaked close to that value (Fig. 7C). The corresponding _1s_CI-Plot peaks within the 68%±*E* range, indicating that the CIs were also correct (Fig. 7D). (The same results without drift correction are shown in Fig. 7E.) However, the _1s_CI-Plot for the 8 nm ruler peaks below 68%-*E* (Fig. 7F), indicating systematic CI errors. The main difference with the simulated data is the presence of high frequency stage fluctuations in the profile of the drift. As these fluctuations represent a real deviation from the quadratic model assumed, we discarded results where the root mean square of the residuals after fitting the drift model exceeded an empirically determined threshold of 0.04 pixels (6.4 nm) (Fig. 7F (*blue*)). The 13 nm distance, however, was unaffected by drift errors, consistent with deviations from the quadratic model of the drift becoming more apparent for shorter separations. We conclude that the FLIP algorithm can accurately measure 8 and 13.3 nm separations, the shorter distance subject to a drift error threshold.

### Resolving two or more Separations

**Figure 8:**
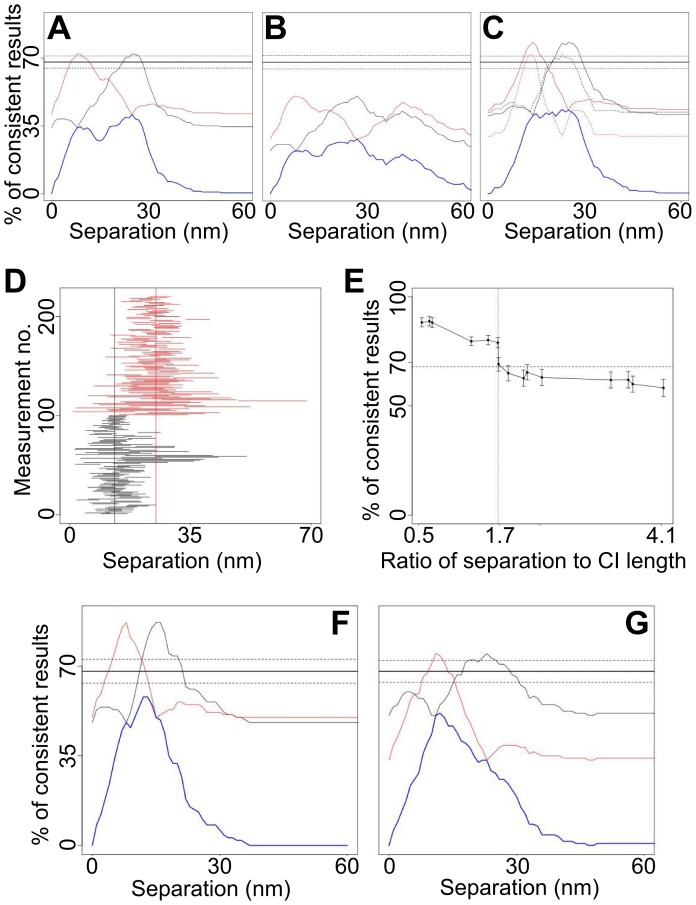
Multiple-distance separations. (A) _1s_CI-Plots (blue) and cross-sections through the _2s_CI-Plots (black and red cross-sections, as shown in Fig. 6*C*) for mixes of simulated datasets with 8 and 25 nm separations. (B) Same as (A) for mixes of 8, 25 and 45 nm separations. (C) Same as (A) for mixes of 13 and 25 nm separations. The solid and dotted lines in correspond to all data and data with CI ≤7 nm respectively. (D) CIs of the simulated mixed 13 nm (black) and 25 nm (red) separations. The six marks show some confidence intervals that miss the correct separation, but hit the other separation. These false positives mean more CIs than expected seem to agree with the 13 nm-25 nm separation pair and the peaks in Fig. 8C exceed 68%. CIs that contain both separations are true positives. (E) Relation between the average CI length and the resolution. For different pairs of distances in simulated data sets, the maximal percentage of consistent measurements in the _2s_CI-Plot is plotted against the ratio of separation to CI length. If the separation of the two distances is small compared to the length of the confidence intervals the CI-Plot has high peaks, indicating that the distances could not be resolved. If the separation between the two distances is larger than 1.7 times the average CI length, then the CI-Plot shows a maximum of around 68% consistent measurements. That indicates that the distances could be resolved. (F) _1s_CI-Plots (blue) and cross-sections through the _2s_CI-Plots (black and red) for mixes of simulated datasets 8 and 13 nm separations and (G) 8, 13 and 25 nm separations. The numbers of datasets with 8, 13, 25 and 45 nm separation were 100, 420, 120 and 60 respectively.

Given that FLIP-like methods (e.g. SHRImP and NALMS) were only applied to population-averaged single-distance measurements, to test the ability of FLIP to resolve two distances we mixed the 8 and 25 nm synthetic data sets of Fig. 7A. The _1s_CI-Plot for this mix peaks below 68% (Fig. 8A, *blue*) and therefore, since we concluded above that the CIs are correct, there must be more than one separation. 68% of the CIs from 8 and 25 nm datasets should agree with 8 and 25 nm separations respectively. In addition, 68% of CIs from the combined data sets should agree with 8 and/or 25 nm and so, as expected, the _2s_CI-Plot peaks at the correct distances and with height 68%±*E* (Fig. 8A, *red and black*). This shows how CI-Plots can be used to compare the hypotheses of one and two separation components. When neither the _1s_CI-Plot nor the _2s_CI-Plot reaches 68%-*E* we must conclude that more than two discrete distances (or a continuum/non-discrete distances) are represented by the data set. This is illustrated by a mix of 8 nm, 25 nm and 45 nm synthetic data sets (Fig. 8B).

To test the resolution limit of this approach, we mixed other synthetic data sets. When mixing 13 and 25 nm data (Fig. 8C) the _1s_CI-Plot is inconsistent with a single distance. Some measurements of the 13 nm separation are not consistent with 13 nm, but are consistent with 25 nm, and vice versa (Fig. 8D). Such measurements artificially increase the agreement, i.e. the height of the peak in the _2s_CI-Plot. Consequently the peak of the _2s_CI-Plot exceeds 68%. This is expected when the difference in separation is comparable to the average CI (<CI>) indicating that the distances cannot be resolved. Since such high peaks can also occur when fitting a larger number of separations than are actually present, no conclusions can be drawn. To improve resolution, we discarded measurements with longer CIs. The peak height fell within the 68%±*E* range when the <CI> was ≤7 nm (Fig. 8*C*, *dashed*). Empirically, we found that this occurs when the two separations are ≥1.7<CI> apart (Fig. 8E), providing the means to predict the separation resolving power of the data.

When mixing 8 and 13 nm data (Fig. 8F), the _1s_CI-Plot is closer to being consistent with one separation while the _2s_CI-Plot peaks higher than before. Because the CIs are not short enough to bring the _2s_CI-Plot peaks into the 68%±*E* range, we cannot determine whether the data is best described by one or two separations and hence they cannot be resolved. To illustrate the effect of mixing unresolved and resolved components we combined the 8, 13 and 25 nm synthetic data (Fig. 8G). The _1s_CI-Plot correctly disagrees with the hypothesis of a single distance but, because 8 and 13 nm separations cannot be resolved, the _2s_CI-Plot incorrectly reports that only two separations are present. We conclude that, unsurprisingly, CI-Plots can only separate distributions into a minimum number of components within the resolution afforded by the CIs.

### Population-averaged Separation Distribution of Inactive EGFRs

**Figure 9:**
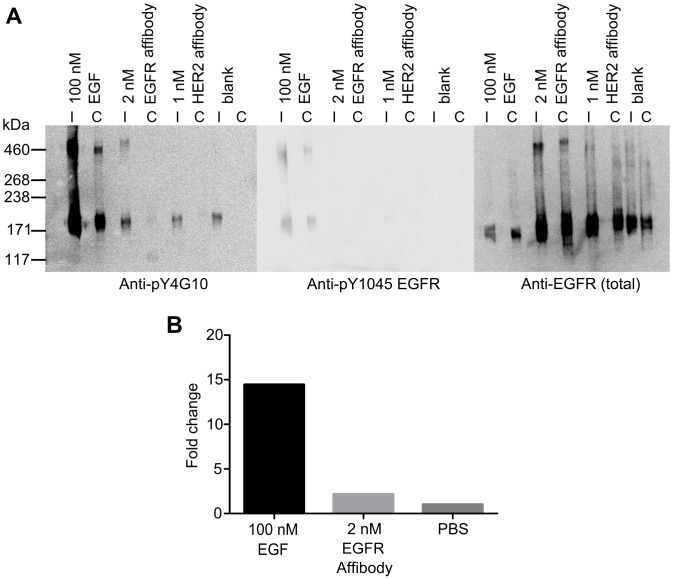
EGFR Affibody molecule does not activate the receptor. (A) Western blots of A431 cell immunoprecipitates (I) and whole cell lysates (C) from unstimulated cells (blank) and cells stimulated with either 100 nM EGF or 2 nM EGFR Affibody. Proteins were probed (left to right) for non-specific tyrosine phosphorylation of proteins (Anti-pY4G10), tyrosine phosphorylation of EGFR (Anti-pY1045 EGFR) to show activated receptor, and presence of EGFR (Anti-EGFR). (B) Results of densitometry normalized against the total EGFR and relative quantity expressed as a fold change compared to an unstimulated control.

We also measured the separations between inactive EGFR in cells. T47D cells were used as models for cells expressing EGFR at low physiological levels (∼7,000 copies). We labelled cells with a 0.5 nM concentration of an anti-EGFR Affibody tagged at a single cysteine residue with the red dye molecule Atto 647N. A concentration titration series suggested that 0.5 nM occupied ∼60% of the surface receptors. We verified by Western Blot that this Affibody does not activate the receptor (Fig. 9).

We collected images from 72 areas in 11 samples. We found more than 40,000 EGFR-Affibody image traces with 5 frames or more. Among these traces, 7.7% did not show any consistent intensity levels, 51.3% showed one intensity level, 21.3% two levels, 10.3% three levels, 5.3% four levels and 4.1% five or more. The presence of multiple steps in the same spot is consistent with receptors being clustered in regions of size comparable to one PSF. Among the 16,740 traces with two or more levels 193 have been selected for FLIP analysis. This was due to crowding and contributions from not smooth backgrounds, which resulted in uneven intensities and/or the intensity of the lowest level dipping below zero. We expect this sample to be representative of the system as a whole because we cannot think of a reason to suspect that the selection of data performed in our analysis has biased the results. For this there would have to be a correlation between the intrinsic receptor separations at the nanoscale and either: the separations of receptors on the >1 µm scale (which is much larger than the size of rafts and signalling platforms [17]); the intensity, blinking and/or bleaching of the fluorophores; and/or variations in the image background fluorescence on a spatial scale similar to the PSF, since these are the key effective selection criteria used in our analysis.

To estimate whether the number of measurements made is sufficient to detect all representative distances, given that the number of values found for each separation in the sample of 193 traces follows a multinomial distribution, we can calculate, for example, that we would observe a separation that is 20% abundant 38.6 times with an error of 5.6, a separation that is 10% abundant 19.3 times (error 4.2), a separation that is 5% abundant 9.7 times (error 3.0) and a separation that is 1% abundant 1.9 times (error 1.4). In other words, the probability of having at least 5 samples of a separation that is 10% abundant is >99.9%, of a separation that is 5% abundant is 96.7%, of a separation that is 2% abundant is 34.4% and of a separation that is 1% abundant is 4.6%.

We were unable to find a significant number of spots with three or more steps in which more than two levels were suitable for analysis. This is unsurprising considering the small fraction of spots with two good levels. This was also the case when other probes were used, like EGF, and in other cells lines, like HeLa cells (unpublished data). We therefore concluded there was no rationale to extend the FLIP algorithm to analyse three or more levels. However, should other experimental conditions allow or require analysis of more than two levels, extension of the bootstrapping method to analyse more than two levels per spot could be easily achieved. One option would be simply to globally fit a 3 (or more) fluorophore model to the data, assessing the CIs using bootstrapping, however this would likely prove computationally impractical. A tractable approach would be to first perform the 2-fluorophore bootstrap analysis, giving a bootstrap distribution of the 2-fluorophore parameters. Bootstrap sample parameters could then be extended to include the next fluorophore/intensity level as follows. To create each new 3-fluorophore sample, the data corresponding to the third intensity level is randomly resampled with replacement, and the model for the first two fluorophores is subtracted using the parameters from a randomly chosen 2-fluorophore sample [***x_1_, y_1_, I_1_, x_2_, y_2_, I_2_, fwhm***], before simply fitting with a Gaussian of fixed width ***fwhm***, to determine the position and intensity of fluorophore 3 [**x_3_, y_3_, I_3_**]. This gives a 3-fluorophore sample [***x_1_, y_1_, I_1_, x_2_***, y***_2_, I_2_, x_3_, y_3_, I_3_, fwhm***]. By creating many such samples, a bootstrap distribution for 3-fluorophores could be obtained which should have most of the benefits of a global fit (mainly missing the effect of uncertainty in fluorophore 3 properties on the properties of fluorophores 1 and 2 which should be small). The process could be repeated to add each further fluorophore.

**Figure 10:**
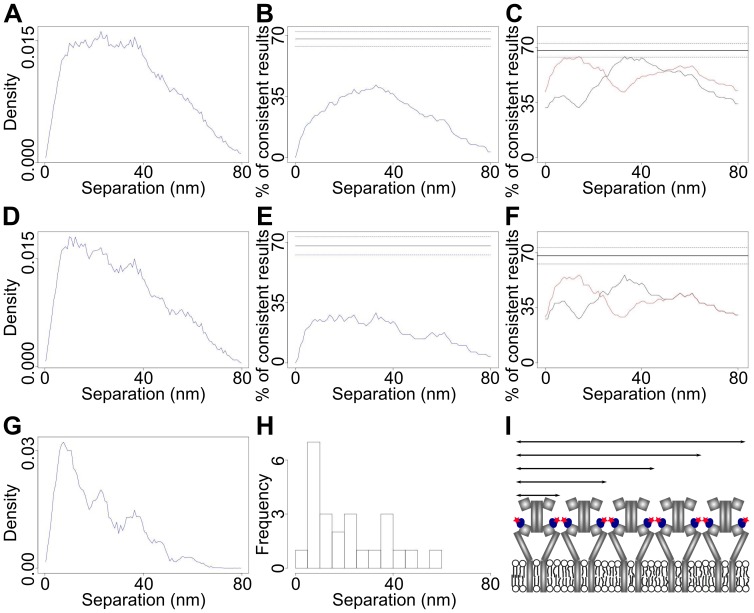
Separations of surface HER1-Affibody complexes in T47D cells. (A) Separation distribution formed by summing 121 individual separation density functions. (B) The corresponding _1s_CI-Plot (blue) and (C) cross-sections through the _2s_CI-Plot (black and red). (D) Separation distribution formed by summing the 79 individual separation density functions with CIs ≤20 nm. (E) The corresponding _1s_CI-Plot (blue) and (F) cross-sections through the _2s_CI-Plot (black and red). (G) Separation distribution formed by summing the 24 individual separation density functions with CIs ≤10 nm. (H) Histogram of best fits for the data in (G). (I) Model of EGFR homo-polymer that can explain the distances derived from (G).

The selection of spots with two good levels happened in two stages, a pre-selection using scores (see Materials and Methods) followed by a visual inspection aided by visualization software [33]. Spots with three or more levels where the lowest two levels passed the selection criteria were also used (FLIP analysis is only possible if the lowest level is good enough). Fig. 10A shows the summed separation density of results with best fit values of ≤80 nm (121 traces) from EGFR-Affibody-Atto647N on T47D cell samples. For this data set we achieved localisation errors down to 1 nm (the median error is 6.1 nm). Because we can calculate the <CI> of these separation data, derived from the CI histogram in Fig. 5B, which is 19 nm, we can conclude that the separation resolving power of these data sets is 1.7<CI> = 32 nm. The presence of a distinct peak in Fig. 10A implies preferred EGFR separations on the surface of T47D cells. Indeed, the _1s_CI-Plot for EGFR-Affibody complexes has a peak height <<68%-*E* (Fig. 10B), while the _2s_CI-Plot cross-sections have peak heights within the range 68%±*E* (Fig. 10C), suggesting that at the resolution of 32 nm we can detect at least two separations in the 0–80 nm range. Given that distances separated from each other less than 32 nm will appear as single distances at this resolution, to test whether more separations were present we introduced an error threshold to improve the resolution of the measurement. For example, Fig. 10D shows a summed separation density in which only the 79 traces with CIs ≤20 nm were included. This reduces the <CI> to 13 nm and increases the separation resolving power to ∼22 nm. Note that the increased resolution has a significant effect on both diagnostic plots. The _1s_CI-Plot (Fig. 10E), which moves further away below 68%, becoming less consistent with the hypothesis of one distance and the _2s_CI-Plot (Fig. 10F) no longer has peak heights within the range 68%±*E* and is therefore no longer consistent with the hypothesis of two distances. These two CI diagnostic plots strongly suggest that the distribution contains more than two distances but that these are still unresolved by the summed separation density with a resolving power of ∼22 nm in Fig. 10D. This information could not be confidently derived without the CI-Plot diagnostics because the increased resolution had a smaller effect in the sum of densities (Fig. 10D). If one continues the process of increasing resolution by imposing an error threshold as small as that used in [28], [29], five peaks were finally clearly distinguished when only separation densities from traces with CIs ≤10 nm were included in the sum of densities (Fig. 10G). This decreased the <CI> and increased the separation resolving power of the distribution to ∼13 nm. For comparison we show the equivalent histogram of best-fit separations for the data in Fig. 10D, from which it was not possible to determine either the number of separations or their values (Fig. 10H). This illustrates the advantage of using a sum of densities to exploit all the information content in the data.

The five peaks in Fig. 10G are vaguely discernable in sum of densities with poorer resolution (Fig. 10A and 10D), which contain many more data, suggesting that no artifacts have been introduced by selecting the spots with the smallest errors. Nevertheless, given that high resolution was achieved at the expense of much fewer data points, we tested that the distribution in Fig. 10G contained a representative sample of the overall population by deriving the 13 nm resolution distribution twice in independent sets of experiments. We found the results to be reproducible.

A simple least squares fit of a sum of five Gaussians to the sum of densities gives peaks at 8 nm, 22 nm, 37 nm, 46 nm and 57 nm. A simple model that may be consistent with our results is shown in Fig. 10I. In this model the receptors are forming a linear polymer that repeats with a periodicity approximately consistent with the value of the distance between two affibodies in the same dimer. The shortest peak may be consistent with the distance one would expect for EGFR dimers from crystallographic data (∼11 nm) [3], [4] if one allows from the contribution to be expected from the very short distances between affibodies side-by-side between adjacent dimers (Fig. 10I). The value of the rest of the distances appears to correlate well with a linear tetramer (∼2×11 nm), a hexamer (∼3×11 nm) an octamer (4×11 nm) and a decamer (5×11 nm).

## Discussion

We have demonstrated a universally applicable SHRIMP/NALMS-variant optimized for measuring separations in the plasma membrane of intact cells. Key advantages of the method are: (i) it has reduced the likelihood of correlated positional errors between the molecules in the spot (Fig. 2B) via the use of a 7-parameter global fit, which is important in cell samples where errors are often comparable to the distances of interest; (ii) it has provided an accurate estimation of the errors via the use of bootstrapping to derive a full separation density function for each distance measured (Fig. 1D) and (iii) it has provided a set of diagnostics, i.e. one-dimensional and two-dimensional distributions of CIs (Fig. 6B and 6C), to derive information on the number of distances in the distribution and to estimate the resolution of the data (Fig. 8E).

Challenges remaining are the larger size and variably of errors intrinsic to images from cells (Fig. 5B), which unavoidably resulted in a smaller percentage of data that can be used for very high resolution measurements when compared to typical data from glass-immobilised molecules (Fig. 5A). Here we substantially mitigated this effect plotting a sum of individual bootstrap densities instead of a histogram of best fits, and by exploiting the individual bootstrap densities in our key quantitative analyses to allow optimal utilisation of all the information content from each separation. Another challenge is the molecular crowdedness on the cell surface, which is ubiquitous even in cells expressing very low number of receptors (Fig. 3B) having the effect of reducing further the number of spots that can be analysed. Crowdedness could in principle be addressed by using image deflation methods in which nearby spots that are too close to a given spot are subtracted before the FLIP algorithm is applied. However, this would unavoidably increase the errors and reduce overall resolution. Given that data selection is therefore hard to avoid in the challenging analysis of single molecule data where often only subsets are analysable, let alone “of interest” [33], it should be noted that any work using this method, or indeed any other which requires some selection of a subset of data, must consider carefully any possibility of biasing the specific conclusions drawn.

We used FLIP to measure the separations between cell surface inactive EGFR-Affibody complexes in the range 0–80 nm (Fig. 10), (Fig. 9). Using a data set with a resolution of ∼13 nm we found that the population-averaged separation distribution contains five components with distance values of about 8 nm, 22 nm, 37 nm, 46 nm and 57 nm (Fig. 10G). Achieving this resolution requires fixation procedures to immobilise cell surface EGFR proteins because the algorithm assumes stationary fluorophores after sample drift has been corrected. Although one can never completely rule out the possibility that chemical cross-linking may introduce artifacts, it is reasonable to assume that receptor topology would have been preserved given that the receptors are on the cell surface and molecular fixation should have been instantaneous. Under this assumption, the novel insight that can be drawn from the data is that, besides monomers and dimers, inactive EGFRs can also exist as ordered arrays of larger oligomers. The argument that the dimerisation/oligomerisation of inactive EGFRs is an artifact introduced by cell over-expression is not valid here because the T47D cells we employed expressed a very small number of receptor molecules. We also believe that any effects from the low temperature used for ligand binding to cells will be small and will not affect the results. This is based on previous work that showed that neither receptor dimerisation, oligomerisation, nor the initial stages of signal transduction are inhibited by the low temperature [38]–[40]. Data collection was carried out at room temperature, which is above the temperature at which a phase transition is known to occur in the plasma membrane of cells [41]. The physical basis for the five distances is currently under investigation outside the scope of this methods paper. However, given that EGFR colocalises with actin filaments, both in control and EGF-stimulated cells [42], and that EGFR directly and specifically binds to polymerized actin [43], possibilities include binding of EGFR to cortical F-actin, which is a single left-handed genetic helix with approximately 13 G-actin molecules repeating every six turns in an axial distance of 35.9 nm [15]. It is therefore tempting to speculate that the separations observed arise from 5 EGFR dimers side-by-side following a cortical F-actin template (Fig. 10I).

The existence of inactive EGFR dimers and oligomers is consistent with some previous data [6]–[11]. It is, however, inconsistent with recent results derived using the number and brightness correlation analysis technique [16], which suggested that inactive EGFR in cells expressing low numbers of receptor copies were monomeric. The discrepancies with our results may be due to the fact that the number and brightness method can only determine the size of macromolecules diffusing over length scales comparable to or larger than the resolution of optical microscope (typically >300 nm). This method would therefore be insensitive to receptors that are either immobile (e.g. in caveolae [40], coated pits [44], or bound to F-actin [43]) and/or that show confined diffusion through length scales comparable to the diffraction-limited resolution of optical microscopy [12], [45], [46].

Given that the formation of a receptor dimer appears to be sufficient for EGFR activation [5], one must question what the possible biological reasons may be for receptors to be in the larger groups. One possibility is efficient detection of environmental cues (such as activating ligands) because, by being in close proximity, the probability of detecting ligand increases with the size of the receptor group. Another possibility is signal amplification. Interestingly, it has been shown that individual ligands can activate several EGFR molecules [47], [48] which would appear to be consistent with the groups of receptors suggested by our data.

In conclusion, we propose that the method presented here will add an important contribution to the tool box utilized by the EGFR signalling community to test a battery of current signal transduction hypotheses and models currently under discussion in the field. Given that dimers, oligomers and clusters are often observed in families of membrane proteins, we also expect that our method will be of interest to any biologist interested in correlating membrane protein structure with function at the plasma membrane, removing a fundamental divide between structural and cell-based work.

## Materials and Methods

### DNA Ruler Sample Preparation

35 mm no. 0 glass-bottomed dishes (MatTek Corporation, USA) were cleaned with piranha solution (3∶1 concentrated sulphuric acid: 30% w/v hydrogen peroxide) and rinsed with water. Dishes were coated with 1 mg/ml BSA-biotin for 10 min at room temperature and rinsed with TE buffer, followed by 0.2 mg/ml streptavidin for 10 min and rinsing. A 0.01 nM solution of Atto 647N- and biotin-labelled 8 nm or 13.3 nm duplex DNA [29] in 10 mg/ml BSA in TE was added for 10 min in the dark. Samples were rinsed with TE and AF3 antifade (Citifluor, UK) added.

### Cell Culture

All reagents were from Invitrogen, UK. T47D cells (ECCAC culture collection) were grown in phenol-red free RPMI 1640 and A431 cells (ECCAC culture collection) in phenol-red free Dulbecco’s Modified Eagles medium (DMEM), both supplemented with 10% (v/v) fetal bovine serum, 2 mM glutamine and 1% penicillin-streptomycin. Poly-L-lysine (Sigma) or Nanogel [49] coated 35 mm no. 1.5 glass-bottomed dishes (MatTek Corporation, USA) were seeded with 2.5×10^5^ cells, grown to ∼70% confluency and serum-starved overnight in serum-free media.

### Cell Labelling

Cells were rinsed with PBS and cooled to 4°C (on ice in the fridge) for 10 min. T47D cells were labelled with 0.5 nM EGFR Affibody-Atto 647N for 30 min at 4°C. The EGFR Affibody was labelled at a 1∶1 ratio at its single cysteine residue. Cells were rinsed with PBS and fixed with 3% paraformaldehyde plus 0.5% glutaraldehyde for 15 min at 4°C then 15 min at room temperature. Cells were fixed to avoid fluorophore movements relative to the cells during image collection.

### Western Blots of Receptor Activation by Affibody Molecules

A431 cells were seeded on 10 cm dishes and 6-well plates, grown to 80% confluence and serum-starved overnight. Cells were treated with 100 nM murine EGF (+ve control), 2 nM EGFR Affibody-Alexa 488 or PBS-BSA for 4 h. After washing off excess label, samples were treated with 1 mg/ml BS3 (Sigma) in PBS for 30 min at 4°C to cross-link proteins. The reaction was quenched with 20 mM Tris pH 7.5 for 15 min on ice and then samples were washed twice with ice-cold PBS. Dishes were lysed for 5 min on ice with 1 ml lysis buffer (50 mM Tris pH 7.5, 5 mM EGTA, 150 mM NaCl, 1% Triton, supplemented with 25 mM benzamidine, 1/100 Protease Inhibitor cocktail (Sigma) and 100 mM NaF, 1 mM Na_3_VO_4_) followed by scraping with a cell scraper. 6-well plate wells were lysed with 200 µl 2x NuPAGE LDS Sample Buffer (Invitrogen), Reducing agent 1x supplemented with 25 mM benzamidine, 1/100 Protease Inhibitor cocktail (Sigma), 100 mM NaF and 1 mM Na_3_VO_4_. Lysates were cleared by centrifuging at 14000 g for 5 min at 4°C.

Protein S resin (Sigma) was washed twice with PBS and incubated with anti-EGFR antibody (Cell Signaling Technology #2256) for 1 h at room temperature, then washed twice with PBS to remove unbound antibody. Lysate was incubated with resin overnight at 4°C and resins were washed four times with TBS-0.1% Triton X-100. Immunoprecipitated proteins were eluted by incubation with 2x NuPAGE LDS Sample Buffer and 10x Sample Reducing Agent (Invitrogen) by boiling for 5 min. Samples were run on 1.5 mm thick 3–8% Tris –Acetate NuPAGE gels (Invitrogen) with HiMark Prestained HMW protein standard (Invitrogen) on a XCell apparatus (Invitrogen). Proteins were blotted using iBlot system (Invitrogen) on nitrocellulose membranes, blocked for 1 h at room temperature with 3% non-fat dry milk in TBS and probed overnight with mouse anti-phosphotyrosine 4G10 (Upstate (Millipore)). Gels were probed with secondary anti-mouse-HRP antibody (Jackson ImmunoResearch) and incubated with Supersignal West Pico Chemiluminescent Substrate solution (Pierce) for 5 min, then imaged with a Kodak Imagestation 4000 MM Pro. Blots were stripped and re-probed sequentially with rabbit anti-EGFR pY1045 (Cell Signaling Technology) and rabbit-anti total EGFR (Cell Signaling Technology). Anti-rabbit HRP (Jackson ImmunoResearch) was used for both blots and images were acquired as above.

Densitometry analysis was performed with ImageJ software (NIH). Bands were normalized against the amount of total EGFR and relative quantity was expressed as fold change compared to the negative control.

### Single-molecule Microscopy

We used an Axiovert 200 M microscope with TIRF illuminator (Zeiss, UK) and incorporating a 100x oil-immersion objective (α-Plan-Fluar, NA = 1.45; Zeiss, UK) and an EMCCD (iXon X3; Andor, UK) Samples were illuminated with ∼0.5 µW/µm2 from an Vortran Stradus 638 nm diode laser (Laser Technology, Inc., USA) and images collected every 0.28 sec.

### Boostrap Calculations

The probability distribution of the 7 parameters inferred from each two-fluorophore spot was assessed using the bootstrap method [35], whereby 1200 new datasets were generated from it by randomly resampling the data, with replacement, and each resampled dataset was then fitted as described above. The distribution of parameters from these fits should reflect the probability distribution of the parameters due to noise in the data, assuming the data comprise a representative sample from their probability distribution. The more frames there are in each level, the more reasonable this assumption becomes. By calculating the lateral separations for each of the bootstrap fitted x and y positions the probability distribution for the fluorophore separation was determined. The quoted measurement and error bar for the each separation measurements is the mean and standard deviation of this probability distribution. Fig 1D shows such an example bootstrap distribution. By evaluating this probability distribution and inferring the measured quantities using this technique we account for correlations between parameters.

### Calculation of the Confidence Interval

Since the distribution of the bootstrapping data is not symmetric there is no canonic way of calculating the CI. We consider both the best fit separation r_best_ and the probability density of each separation p(r). The density was calculated using kernel density estimation with a Gaussian kernel and Silverman’s rule of thumb bandwidth estimation. We assigned a weighted distance to each bootstrapping sample separation r: d(r, r_best_) = | r - r_best_|/p(r). The 68% confidence interval comprises the 68% bootstrapping sample separations with the shortest weighted distance to the best fit value.

### Pre-selection of Appropriate Traces

Tracks are formed by linking detected features between frames, with gaps due to blinking or limitation of the detection method filled by interpolating linearly in position [30]. To find appropriate tracks for separation measurements we calculate a number of statistics and scores for each track whose values we use to pre-select tracks by according to empirically determined thresholds [30]. The pre-selected traces are further filtered by eye. We have chosen the following criteria:

Good agreement of the levels intensity trace with the level model (using a χ^2^ statistic)

At least two non-zero intensity levels in the trace

At least five non-interpolated frames in each level

The trace bleaches completely during acquisition

At least 50% of features in a trace detected rather than interpolated

Minimum distance to closest neighbour of 2 pixels = 320 nm

Distance of the mean position of features in level 1 and features in level 2 (<112 nm); the distance roughly is half the expected separation of the fluorophores

At least 50% of frames in a trace that are assigned to a constant intensity level

A standard error in mean criterion for the distance of the mean positions in level 1 and level 2

A homogeneity (compactness of intensities) and separation criterion for the intensity of the features in the levels.

### Filtering of Measurements

The CI length filter criterion is compared with the difference of the longest and shortest separation of a CI. For the distance filtering we required that the probability (based on the kernel density estimation) that a measurement fulfils the filter criterion is at least 75%.
